# An Externally Validated Dynamic Nomogram for Predicting Unfavorable Prognosis in Patients With Aneurysmal Subarachnoid Hemorrhage

**DOI:** 10.3389/fneur.2021.683051

**Published:** 2021-08-26

**Authors:** Ping Hu, Yang Xu, Yangfan Liu, Yuntao Li, Liguo Ye, Si Zhang, Xinyi Zhu, Yangzhi Qi, Huikai Zhang, Qian Sun, Yixuan Wang, Gang Deng, Qianxue Chen

**Affiliations:** ^1^Department of Neurosurgery, Renmin Hospital of Wuhan University, Wuhan, China; ^2^Department of Neurosurgery, the Affiliated Hospital of Panzhihua University, Panzhihua, China

**Keywords:** aneurysmal subarachnoid hemorrhage, unfavorable prognosis, LASSO regression, multivariable logistic regression, dynamic nomogram, external validation

## Abstract

**Background:** Aneurysmal subarachnoid hemorrhage (aSAH) leads to severe disability and functional dependence. However, no reliable method exists to predict the clinical prognosis after aSAH. Thus, this study aimed to develop a web-based dynamic nomogram to precisely evaluate the risk of poor outcomes in patients with aSAH.

**Methods:** Clinical patient data were retrospectively analyzed at two medical centers. One center with 126 patients was used to develop the model. Least absolute shrinkage and selection operator (LASSO) analysis was used to select the optimal variables. Multivariable logistic regression was applied to identify independent prognostic factors and construct a nomogram based on the selected variables. The C-index and Hosmer–Lemeshow *p*-value and Brier score was used to reflect the discrimination and calibration capacities of the model. Receiver operating characteristic curve and calibration curve (1,000 bootstrap resamples) were generated for internal validation, while another center with 84 patients was used to validate the model externally. Decision curve analysis (DCA) and clinical impact curves (CICs) were used to evaluate the clinical usefulness of the nomogram.

**Results:** Unfavorable prognosis was observed in 46 (37%) patients in the training cohort and 24 (29%) patients in the external validation cohort. The independent prognostic factors of the nomogram, including neutrophil-to-lymphocyte ratio (NLR) (*p* = 0.005), World Federation of Neurosurgical Societies (WFNS) grade (*p* = 0.002), and delayed cerebral ischemia (DCI) (*p* = 0.0003), were identified using LASSO and multivariable logistic regression. A dynamic nomogram (https://hu-ping.shinyapps.io/DynNomapp/) was developed. The nomogram model demonstrated excellent discrimination, with a bias-corrected C-index of 0.85, and calibration capacities (Hosmer–Lemeshow *p*-value, 0.412; Brier score, 0.12) in the training cohort. Application of the model to the external validation cohort yielded a C-index of 0.84 and a Brier score of 0.13. Both DCA and CIC showed a superior overall net benefit over the entire range of threshold probabilities.

**Conclusion:** This study identified that NLR on admission, WFNS grade, and DCI independently predicted unfavorable prognosis in patients with aSAH. These factors were used to develop a web-based dynamic nomogram application to calculate the precise probability of a poor patient outcome. This tool will benefit personalized treatment and patient management and help neurosurgeons make better clinical decisions.

## Introduction

Aneurysmal subarachnoid hemorrhage (aSAH) is an acute cerebrovascular injury with high morbidity and mortality worldwide (1). Roughly one-third of survivors have severe disability and functional dependence, some of which may be predicted and treated early (2, 3). Therefore, a practical prediction model for treating and evaluating these patients with aSAH is urgently needed.

Several studies have shown that a series of serum biochemical and immunological indices could predict the prognosis of patients with aSAH after surgery, including white blood cell (WBC) count, neutrophil-to-lymphocyte ratio (NLR), C-reactive protein level, and blood glucose level on admission (4–6). Meanwhile, predictive models combining multiple independent predictors have been used to predict prognosis. However, these predictive models lack calibration tests, model external validation, or evaluation of their clinical usefulness; therefore, the accuracy and practicability of the models are difficult to assess (7, 8). High-quality predictive models are necessary to guide clinical decision-making, personal care, and patient management (9). Therefore, a more practical and accurate predictive model is urgently needed in this field. A web-based dynamic nomogram that precisely calculates the probability of the disease is a more precise and practical tool than standard nomograms and some predictive models (10, 11). However, no study has reported a dynamic nomogram to predict the clinical prognosis after aSAH.

Thus, this study aimed to develop a dynamic web-based nomogram that incorporated blood laboratory tests, clinical state at admission, and postoperative complications for the prognosis of patients with aSAH. The predictive model was validated using internal and independent external validation cohorts.

## Materials and Methods

### Study Design and Patient Enrollment

This retrospective observational cohort study was conducted in two centers. A training cohort of 126 patients with aSAH treated in the Department of Neurosurgery, Renmin Hospital of Wuhan University, from September 1, 2019 to September 1, 2020, was used to develop the model. A total of 84 patients admitted to the Department of Neurosurgery, the Affiliated Hospital of Panzhihua University, from September 1, 2019 to September 1, 2020, were used to validate the model. The diagnosis of aSAH was assessed by head CT, CT angiography, or digital subtraction angiography according to the guidelines (12, 13).

The exclusion criteria were as follows: (1) non-aneurysmal or traumatic SAH; (2) clinical history of aSAH for more than 48 h; (3) complicated with vascular anomalies and malformations; (4) complicated with intracerebral hemorrhage; (5) taking anticoagulants or corticosteroids within 1 month of hospitalization; (6) postoperative state on admission; (7) no surgical treatment performed within 2 days of onset; (8) acute infection on admission; (9) other organs with extreme dysfunction; (10) bilateral mydriasis or other permanent brain injuries on admission; (11) missing data.

### Data Collection

Clinical information including demographic data (admission number, name, sex, age, hypertension, smoking, and alcohol consumption), clinical state, radiological findings, serum laboratory tests (glucose, D-dimer, WBCs, neutrophils, lymphocytes, and monocytes), and CT Hounsfield unit (HU) values on admission were collected using an electronic medical record system. The number, location length, and neck size of the aneurysms were also recorded. Hunt and Hess classification and the World Federation of Neurosurgical Societies (WFNS) were used to measure the clinical characteristics and neurological status at admission. The radiological features at admission were classified according to the modified Fisher grade of the CT scan (14–16).

All patients were treated with either a surgical clipping or coil embolization as soon as possible. The postoperative routine therapies included hemostasis, analgesics, anti-inflammatory drugs, and nimodipine for anti-vasospasm. An immediate postoperative head CT scan was performed to identify intracranial rebleeding or cerebral infarction after the operation.

### Admission CT Values

The mean CT values of the blood clots in the subarachnoid space were measured. The regions of interest were manually drawn on representative slices by two neurosurgeons who were blinded to the patients' clinical characteristics. The density of blood clotting in the subarachnoid space on CT slices was assessed using the mean HU value. The following subarachnoid cisterns/fissures were used to measure the mean HU: lateral Sylvian fissures, anterior interhemispheric fissures, medial Sylvian fissures, suprasellar cistern, ambient cistern, and quadrigeminal cistern, as previously described (17, 18).

### The Definition of Delayed Cerebral Ischemia

Delayed cerebral ischemia (DCI) was identified as follows: (1) CT scan omitting other causes of focal neurological decline such as aphasia, apraxia, hemianopia, or neglect, either permanent or temporary, within 4 to 14 days after SAH; (2) a Glasgow Coma Scale decrease of at least 2 points lasting for at least 1 h and not immediately evident after surgery; (3) head CT scan revealing a new cerebral infarction within 4–30 days after aSAH, which was not noticeable on admission or immediately after the operation, and no other explanation except for vasospasm (17, 19).

### Outcome Assessment

Three months after the initial bleeding, the functional outcome was dichotomized as excellent or poor according to the modified Rankin Scale (mRS) (20–22). A favorable outcome was defined as a score of 0–2 (perform activities without assistance) on the mRS, while a score of 3–6 (moderate to severe disability or death) was defined as a poor outcome. The results were evaluated via telephone by a neurosurgeon who was blinded to the clinical and imaging data.

### Sample Size

In the current literature, the events per variable (EPV) criterion, notably an EPV of 10, is widely applied as the lower limit for developing logistic regression models that predict a binary outcome (23, 24). Thus, an EPV of 10 was used to estimate the effective sample size in this study. A total of three variables, which were incorporated into the multivariate logistic analysis, were selected by least absolute shrinkage and selection operator (LASSO) regression analysis. Thus, the effective sample size was at least 30 in the training cohort.

### Statistical Analysis

Statistical analysis was performed using IBM SPSS Statistics for Windows, version 26.0 (IBM Corp., Armonk, NY, USA) and R software (https://www.r-project.org/). Continuous variables were analyzed using independent *t*-test or Mann–Whitney U test and are presented as means ± SD or medians with interquartile range. Categorical variables are expressed as numbers (percentages) and were analyzed using χ^2^ or Fisher exact tests. Based on the limited sample size in this study, LASSO regression, which is suitable for analyzing high-dimensional data, was used to select the most informative prognostic variables from the data cohort (25–27). The variables were then entered into a multivariable logistic regression model; a nomogram model was then constructed by integrating independent factors.

The discrimination and calibration capacities evaluated the performance of the nomogram model. Receiver operating characteristic (ROC) curve and area under the curve (AUC) analyses were performed to assess the discriminatory capabilities, while the bias-corrected C-index was calculated using 1,000 bootstraps. The Hosmer–Lemeshow test, Brier score, and calibration curve were used to assess the calibration ability (28). In addition to internal validation, the model performance was also assessed using an external validation cohort. The clinical effectiveness of the nomogram was evaluated by decision curve analysis (DCA) and clinical impact curve (CIC) (29, 30).

We used the “glmnet, corrplot, caret package” in R software to generate the LASSO regression results and the “rms package” to establish the nomogram. The “pROC,” “rmda,” “MASS,” “survival,” “ggplot2,” “ggridges,” “plotROC,” and “riskRegression” packages were applied to generate the C-index, ROC, DCA, CIC, and calibration curve. The “shinyapps.io” and “DynNom packages” were used to develop a web-based dynamic nomogram application to predict unfavorable prognosis, which could precisely calculate the risk probability for unfavorable prognosis at 3 months after aSAH. All tests were two-tailed, and *p*-values < 0.05 were considered statistically significant.

## Results

### Baseline Cohort Characteristics

This study included a total of 210 patients with aSAH, with 126 and 84 patients in the training and external validation cohorts, respectively. The baseline characteristics of the two cohorts were shown in Table 1. Women comprised 137 (65%) patients in the two cohorts, and the mean age was 56.50 years (interquartile range: 53.00, 63.00). We observed no significant differences between the training and external validation cohorts, except for higher admission glucose level, percentages of DCI occurrence and WFNS grade IV–V, and lower aneurysm neck size in the training cohort. The numbers of patients with poor prognoses in the two cohorts, 46 (37%) and 24 patients (29%), respectively, did not differ significantly. Table 2 showed the baseline characteristics of the model development cohort.

**Table 1:** Baseline characteristics of the training and external validation cohorts.

Characteristics^*^	**Training cohort** **(*n* = 126)**	**External validation cohort** **(*n* = 84)**	***P*** **-value**
Demographics			
Age (years)	57.50 (52.25, 63.75)	55.00 (53.00, 58.00)	0.146
Gender (female)	84 (67)	53 (63)	0.594
Medical history			
Hypertension	69 (55)	38 (45)	0.226
Smoking history	28 (22)	14 (17)	0.418
Alcohol consumption	15 (12)	4 (5)	0.128
Admission laboratory			
results			
Glucose (mmol/L)	6.56 (5.56, 7.90)	6.87 (6.18, 8.14)	0.048
D-dimer (mg/L)	1.51 (0.76, 3.65)	0.84 (0.48, 1.80)	<0.001
WBC (10^9^/L)	12.10 ± 3.67	11.55 ± 3.16	0.254
Neutrophil (10^9^/L)	10.09 ± 3.60	9.80 ± 3.05	0.529
Lymphocyte (10^9^/L)	0.85 (0.68, 1.17)	0.94 (0.68, 1.21)	0.489
Monocytes (10^9^/L)	0.56 (0.37, 0.79)	0.58 (0.37, 0.74)	0.725
NLR (10^9^/L)	11.90 (7.77, 17.02)	10.19 (7.77, 16.57)	0.413
Admission CT HU value			
SAH mean HU value	57.22 (52.16, 63.08)	54.85 (51.53, 61.52)	0.099
WFNS grade			0.003
I–III	86 (68)	73 (87)	
IV–V	40 (32)	11 (13)	
Hunt and Hess grade			0.159
I–III	104 (83)	76 (90)	
IV–V	22 (17)	8 (10)	
Modified Fisher scale			0.176
0–2	30 (24)	28 (33)	
3–4	96 (76)	56 (67)	
Aneurysm location			0.404
ACA	7 (6)	4 (5)	
MCA	31 (25)	18 (21)	
ICA	26 (21)	12 (14)	
PCA	1 (1)	2 (2)	
ACoA	30 (24)	22 (26)	
PCoA	20 (16)	22 (26)	
Other	11 (9)	24 (5)	
Aneurysm number			0.443
Single	110 (87)	77 (92)	
Multiple (≥2)	16 (13)	7 (8)	
Mean aneurysm size			
Neck (mm)	3.20 (2.70, 3.70)	3.53 (3.00, 4.62)	0.004
Length (mm)	4.95 (4.00, 6.50)	5.20 (4.00, 6.25)	0.635
Aneurysm treatment			0.832
Clipping	87 (69)	56 (67)	
Coiling	39 (31)	28 (33)	
Hydrocephalus	18 (14)	6 (7)	0.170
DCI	44 (35)	13 (15)	0.003
Poor prognosis	46 (37)	24 (29)	0.296

*aSAH, aneurysmal subarachnoid hemorrhage; WBC, white blood cell; NLR, neutrophil-to-lymphocyte ratio; WFNS, World Federation of Neurosurgical Surgeons; ACA, anterior cerebral artery; MCA, middle cerebral artery, ICA, internal cerebral artery; PCA, posterior cerebral artery; ACOM, anterior communicating artery; PCoA, posterior communicating artery; Other aneurysms include basilar artery, posterior inferior cerebellar artery, and vertebral artery;DCI, delayed cerebral ischemia*.

* *Values are presented as the number of patients (%) unless indicated otherwise*.

**Table 2:** Baseline characteristics of the training cohort according to the prognostic outcomes 3 months after aSAH.

**Characteristics**	**Total** **(***n*** = 126)**	**Favorable** **(***n*** = 80)**	**Unfavorable** **(***n*** = 46)**	***P*** **-value**
Demographics				
Age (years)	57.57 ± 9.76	56.81 ± 8.66	58.89 ± 11.41	0.288
Gender (female)	84 (67)	56 (70)	28 (61)	0.395
Medical history				
Hypertension	69 (55)	45 (56)	24 (52)	0.797
Smoking history	28 (22)	16 (20)	12 (26)	0.570
Alcohol consumption	15 (12)	9 (11)	6 (13)	0.989
Admission laboratory results				
Glucose (mmol/L)	6.56 (5.56, 7.90)	6.16 (5.34, 7.21)	7.04 (6.23, 8.54)	0.002
D-dimer (mg/L)	1.51 (0.76, 3.65)	1.42 (0.79, 2.84)	1.84 (0.74, 5.85)	0.238
WBC (10^9^/L)	12.10 ± 3.67	10.78 ± 2.83	14.39 ± 3.86	<0.001
Neutrophil (10^9^/L)	10.09 ± 3.60	8.94 ± 2.72	12.08 ± 4.07	<0.001
Lymphocyte (10^9^/L)	0.85 (0.68, 1.17)	0.97 (0.73, 1.31)	0.77 (0.53, 0.93)	<0.001
Monocytes (10^9^/L)	0.56 (0.37, 0.79)	0.54 (0.38, 0.70)	0.65 (0.35, 0.95)	0.221
NLR (10^9^/L)	12.63 ± 6.72	10.06 ± 5.15	17.11 ± 6.83	<0.001
Admission CT HU value				
SAH mean HU value	57.22 (52.16, 63.08)	55.28 (51.34, 60.85)	61.43 (55.58, 64.94)	<0.001
WFNS grade				<0.001
I–III	86 (68)	70 (88)	16 (35)	
IV–V	40 (32)	10 (12)	30 (65)	
Hunt and Hess grade				<0.001
I–III	104 (83)	75 (94)	29 (63)	
IV–V	22 (17)	5 (6)	17 (37)	
Modified Fisher scale				0.134
0–2	30 (24)	23 (29)	7 (15)	
3–4	96 (76)	57 (71)	39 (85)	
Aneurysm location				0.493
ACA	7 (6)	5 (6)	2 (4)	
MCA	31 (25)	17 (21)	14 (30)	
ICA	1 (1)	1 (1)	0 (0)	
PCA	26 (21)	20 (25)	6 (13)	
ACoA	30 (24)	16 (20)	14 (30)	
PCoA	20 (16)	13 (16)	7 (15)	
Other	11 (9)	8 (10)	3 (7)	
Aneurysm number				0.010
Single	110 (87)	75 (94)	35 (76)	
Multiple (≥2)	16 (13)	5 (6)	11 (24)	
Mean aneurysm size				
Neck (mm)	3.20 (2.70, 3.70)	3.15 (2.58, 3.93)	3.20 (2.90, 3.60)	0.551
Length (mm)	4.95 (4.00, 6.50)	4.95 (3.90, 6.15)	4.95 (4.04, 6.50)	0.736
Aneurysm treatment				0.273
Clipping	87 (69)	52 (65)	35 (76)	
Coiling	39 (31)	28 (35)	11 (24)	
Hydrocephalus	18 (14)	7 (9)	11 (24)	0.038
DCI	44 (35)	12 (15)	32 (70)	<0.001

*aSAH, aneurysmal subarachnoid hemorrhage; WBC, white blood cell; NLR, neutrophil-to-lymphocyte ratio; WFNS, World Federation of Neurosurgical Surgeons; ACA, anterior cerebral artery; MCA, middle cerebral artery; ICA, internal cerebral artery; PCA, posterior cerebral artery; ACOM, anterior communicating artery; PCoA, posterior communicating artery; Other aneurysms include basilar artery, posterior inferior cerebellar artery, and vertebral artery; DCI, delayed cerebral ischemia*.

### Variable Selection

**Figure 1:**
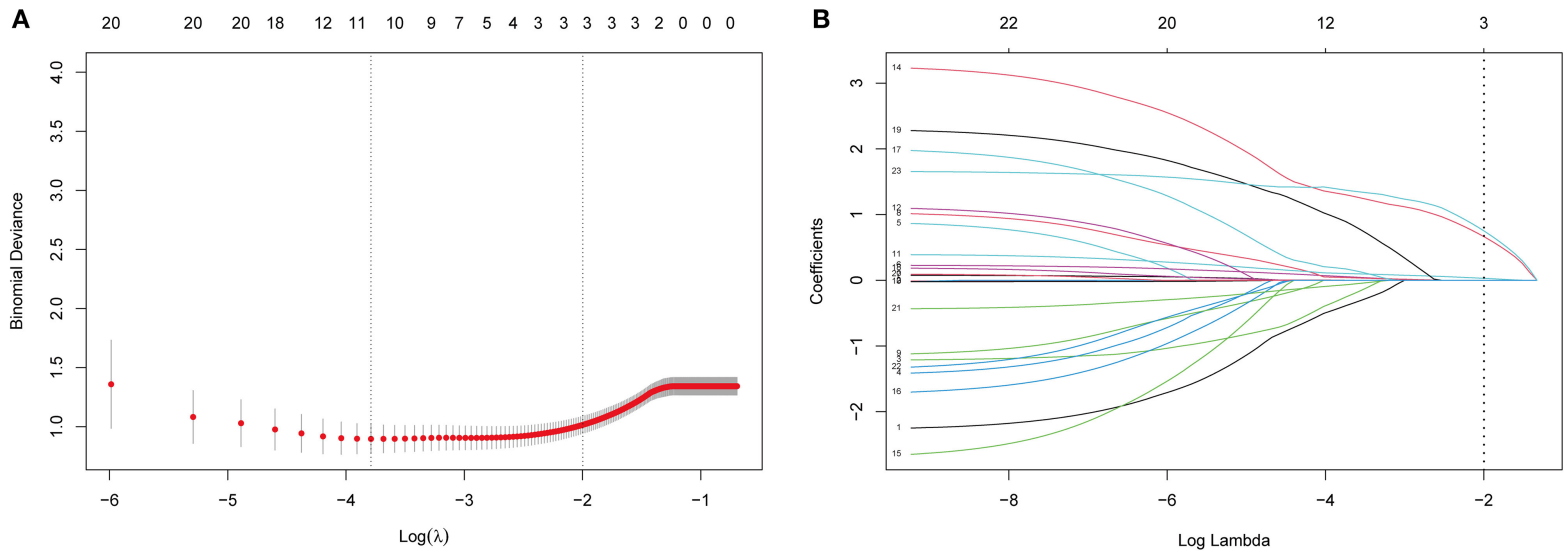
Selection of prognostic variables by least absolute shrinkage and selection operator (LASSO) regression analysis. **(A)** The selection of optimal parameters (lambda) by 5-fold cross-validation. The left and right dotted vertical lines, respectively, represent the optimal lambda values when using the minimum error criterion and one standard error (1-SE) of the minimum criterion. **(B)** The vertical line was plotted at the optimal λ of 0.1356, with log(λ) = −1.997. Three factors with non-zero coefficients were finally selected.

LASSO regression was used to select the most useful variables. As shown in Figure 1A, when using the minimum error criterion, 21 variables were decreased to 11 variables and further reduced to three variables after applying the one standard error (1-SE) criterion. Thus, an optimal λ of 0.1356, with log(λ) = −1.997, was adopted. Three factors with non-zero coefficients were finally selected by 5-fold cross-validation to prevent overfitting (Figure 1B). After adjusting by multivariable logistic regression, NLR [adjusted odds ratio (aOR), 1.143; 95% CI 1.045–1.263, *p* = 0.005], WFNS grade (aOR: 5.025, 95% CI 1.777–14.75, *p* = 0.002), and DCI (aOR: 6.143, 95% CI 2.374–18.13, *p* = 0.0003) were independent prognostic factors (Table 3).

**Table 3:** Multivariable logistic analysis for variables selected by LASSO.

**Variables**	**Coefficient**	**SE**	**aOR (95% CI)**	***P*** **-value**
NLR	0.134	0.047	1.143 (1.045–1.263)	**0.005**
WFNS grade	1.614	0.535	5.025 (1.777–14.75)	**0.002**
DCI	1.858	0.515	6.143 (2.374–18.13)	**0.0003**

*NLR, neutrophil-to-lymphocyte ratio; WFNS, World Federation of Neurosurgical Surgeons; DCI, delayed cerebral ischemia; aOR, adjusted odds ratio; LASSO, least absolute shrinkage and selection operator*.

*Boldface type indicates statistical significance (p < 0.05) by multivariable logistic regression*.

### Prognostic Model Development

**Figure 2:**
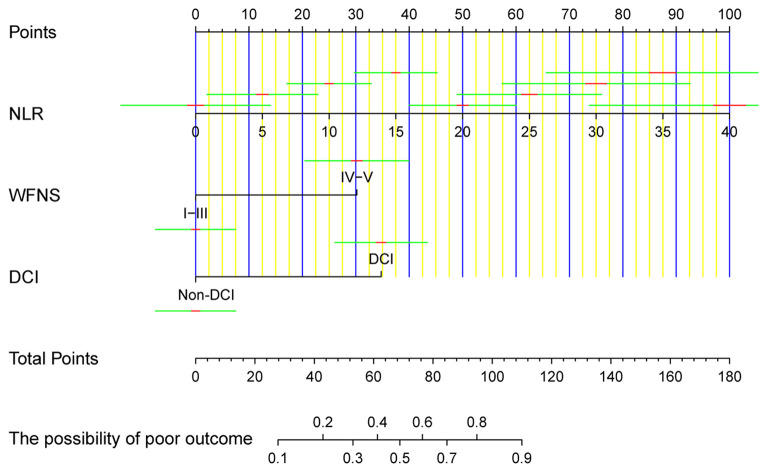
The ordinary nomogram predicts the risk of unfavorable prognosis 3 months after aSAH, based on NLR, WFNS grade, and DCI. NLR, neutrophil-to-lymphocyte ratio; WFNS, World Federation of Neurosurgical Societies; DCI, delayed cerebral ischemia.

All independent factors were used to construct a nomogram to predict unfavorable prognosis 3 months after aSAH (Figure 2). According to the patient's information on admission, each of the three prognostic factors in the nomogram was projected upward to a point. NLR had continuous values ranging from 0 to 100. The WFNS grade was divided into two levels (I–III and IV–V), while DCI was divided into non-DCI and DCI, each of which were assigned points. The total sum of the points from the three variables was converted into an individual poor prognosis risk, in which the higher was the total score, the higher was the risk of an unfavorable prognosis. Based on the ordinary nomogram, we developed a dynamic nomogram web-based application (https://hu-ping.shinyapps.io/DynNomapp/) to precisely calculate the risk probability for unfavorable prognosis at 3 months after aSAH.

### Nomogram Validation

**Figure 3:**
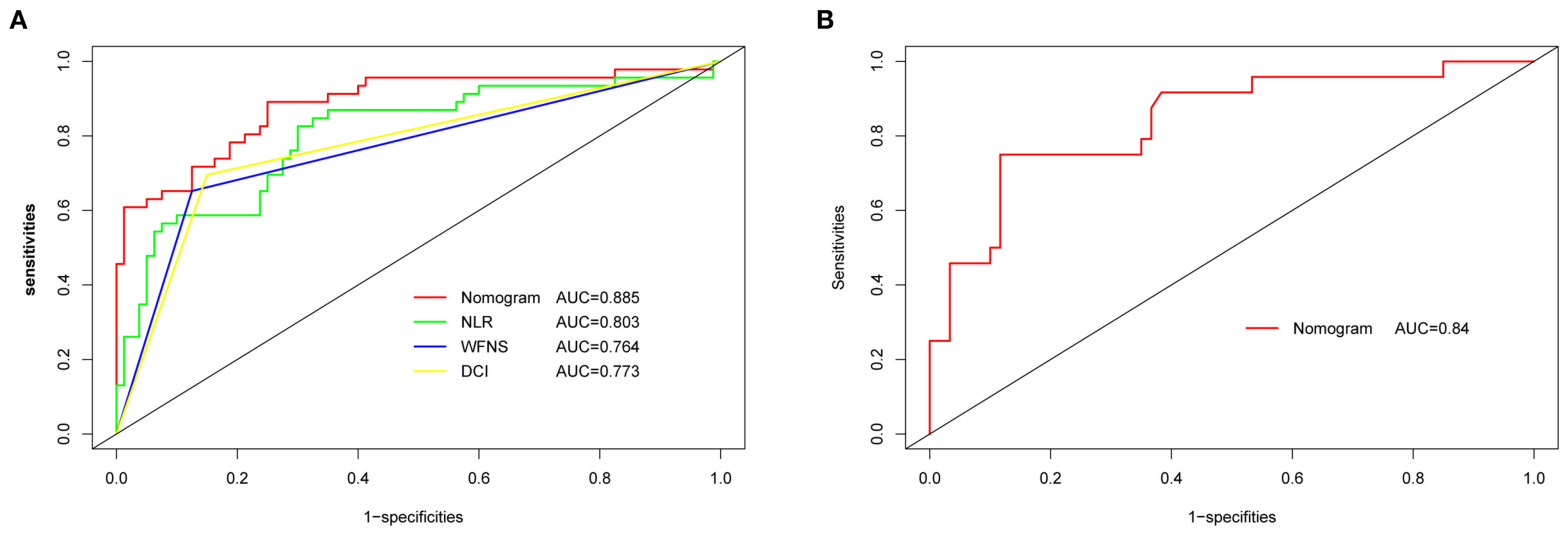
ROC and AUC analysis for nomogram validation. **(A)** Internal validation. **(B)** External validation. AUC, area under the curve; ROC, receiver operating characteristic; NLR, neutrophil-to-lymphocyte ratio; WFNS, World Federation of Neurosurgical Societies; DCI, delayed cerebral ischemia.

**Figure 4:**
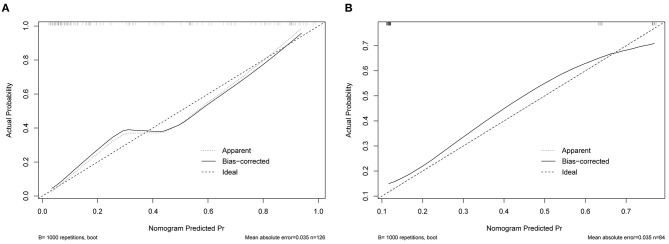
Calibration curve for nomogram validation. **(A)** Internal validation. **(B)** External validation.

The original AUC was 0.88 (95% CI 0.82–0.95), while the bias-corrected C-index with 1,000 bootstraps was 0.82, suggesting that the model had excellent discrimination. Compared to a single independent prognostic factor, the nomogram model better predicted the occurrence of DCI (Figure 3A). The calibration capacity was also internally validated; the Hosmer–Lemeshow *p*-value of 0.41 in the training cohort suggested an excellent fitting of the nomogram. By contrast, a Brier score of 0.12 and a calibration curve with 1,000 bootstrap resamples showed that the model has a good calibration ability (Figure 4A), demonstrating no significant deviation between the actual and predicted probabilities.

**Figure 5:**
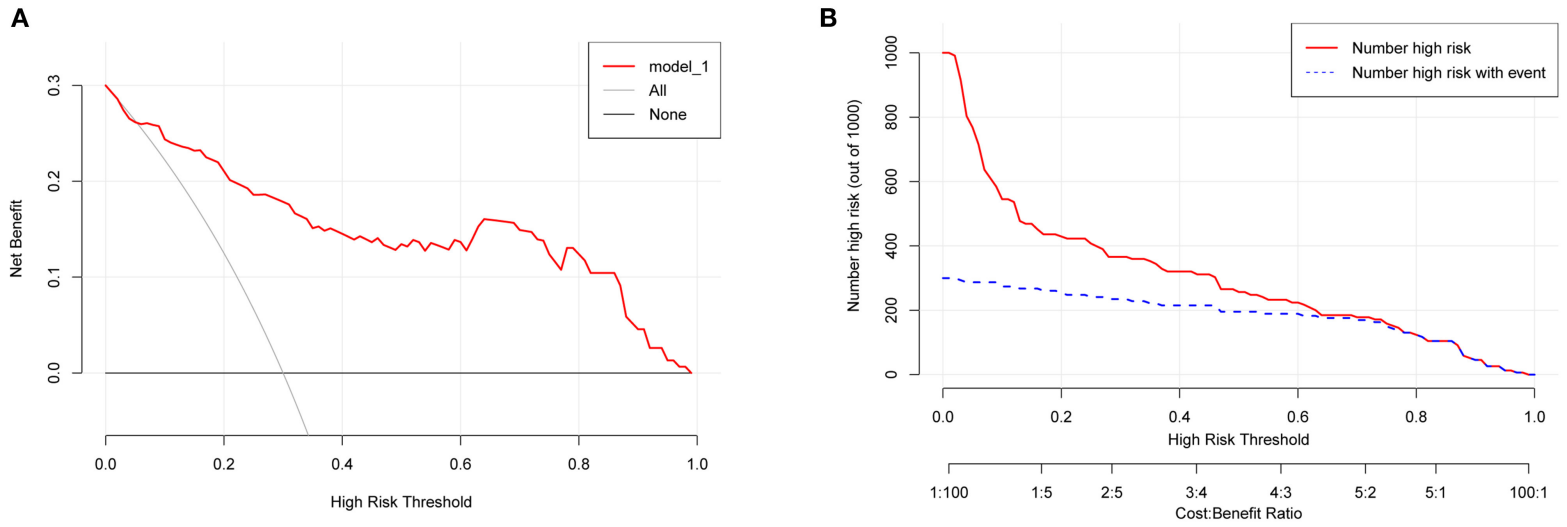
DCA and CIC curves. **(A)** DCA curve. **(B)** CIC curve. DCA, decision curve analysis; CIC, clinical impact curve.

A total of 84 patients retrospectively collected from September 2019 to September 2020 at another center were used for external validation of the model. The validation AUC value of 0.84 (95% CI 0.74–0.94) was consistent with the original AUC value (0.88) in the training cohort (Figure 3B). A Brier score of 0.13 and the calibration curve plotted for external validation showed good calibration of the model in the external validation cohort (Figure 4B). Furthermore, DCA and CIC were used to evaluate the clinical usefulness of our prognostic nomogram. DCA showed a superior overall net benefit for a threshold probability of 0–1 (Figure 5A). CIC also demonstrated good performance over the entire range of threshold probabilities (Figure 5B).

## Discussion

This study constructed and validated a dynamic nomogram model to predict unfavorable prognosis at 3 months after aSAH. The nomogram incorporated the NLR, admission WFNS grade, and DCI. To our knowledge, this study is the first to develop a web-based dynamic nomogram application to calculate the risk of poor prognosis (mRS, 3–6 points). In addition, external validation using data from another center was conducted for the first time. Precise calculation of the probability of the disease makes the dynamic nomogram a more accurate and practical tool compared to ordinary predictive models.

In a previous study, Zhang et al. developed an ordinary nomogram combined with a systemic inflammation response index and other conventional factors to predict the prognostic outcome in patients with aSAH (8). These factors were selected using univariate and multivariate logistic analyses. Lai et al. conducted a predicting model for aSAH prognosis, which included the GCS score, surgical clipping, and NLR (7). These independent prognostic variables were also selected by multivariate logistic regression. Compared with the study by Zhang et al., the model by Lai et al. was only internally validated rather than externally validated. Although a large-scale retrospective study has been conducted on poor recovery in patients with aSAH, the study only contained an internal validation (31). Our study included a total of 84 patients retrospectively collected from September 2019 to September 2020 from another center for external validation of the model. The validation AUC value was nearly identical to the original AUC value in the training cohort. The calibration curve showed good calibration of the model in the external validation cohort.

Contrary to the conventional multivariate logistic method, this study applied the LASSO regression analysis, which performed well in reducing the data dimensionality and decreasing multicollinearity between variables, and it was used to select variables for multivariable logistic regression by minimizing coefficients and reducing variance (32). As a result, an optimal λ of 0.1356, with log(λ) = −1.997, was adapted. Three factors with non-zero coefficients were finally selected by 5-fold cross-validation to prevent overfitting. A logistic regression model was then used to establish a nomogram. The original AUC was 0.88, while the bias-corrected C-index with 1,000 bootstraps was 0.82, suggesting that the model had excellent discrimination. Compared with a single independent prognostic factor, the nomogram model better predicted the occurrence of DCI. The calibration capacity was also internally validated; the Hosmer–Lemeshow *p*-value in the training cohort suggested an excellent fit of the nomogram. By contrast, a Brier score and calibration curve with 1,000 bootstrap resamples showed that the model had a good calibration ability, demonstrating no significant deviation between the actual and predicted probabilities. In addition, DCA showed a superior overall net benefit for a threshold probability of 0–1. The CIC also demonstrated a good performance over the entire range of threshold probabilities.

We observed no significant difference in NLR between the training and external validation cohorts. However, the NLR in patients with an unfavorable prognosis was higher than that in patients with a favorable prognosis in the training cohort (*p* = 0.005). Our study identified NLR on admission as an independent risk factor for unfavorable prognosis, similar to previous findings (4, 7, 33, 34). Lai et al. reported that a high NLR on admission could increase the risk of poor prognosis 3 months postoperatively. Giede-Jeppe et al. suggested that higher NLR was associated with more significant neurological injury, as reflected by higher subarachnoid and intraventricular blood. Therefore, increased NLR resulted in worse functional outcomes beyond these associations. The following possible mechanisms may explain why high NLR on admission increased the risk of unfavorable prognosis at 3 months after aSAH. First, a high NLR could promote the synthesis and secretion of matrix metalloproteinase, which could degrade components of the extracellular matrix and play an essential role in the blood–brain barrier, resulting in secondary brain injury (35, 36). Second, increased NLR at admission may include excessive neuroinflammation caused by elevated neutrophil granulocyte counts and lymphocyte depletion leading to immunodepression. Physiological stress responses lead to an increased postictal release of endogenous catecholamines and cortisol, resulting in secondary brain injury and possibly inflammatory complications (37, 38).

The WFNS grade or GCS have gradually become essential validated scales to evaluate clinical characteristics and neurological status on admission. A good grade was defined as I–III on WFNS, while IV–V on the WFNS was considered poor (39, 40). In our study, 40 (32%) and 11 (13%) patients in the training and external validation cohorts, respectively, had WFNS grades of IV–V. WFNS grade (*p* = 0.002) on admission independently predicted unfavorable prognosis and patients with WFNS grades IV–V had a higher risk of developing a poor prognosis compared with patients with WFNS grades I–III. This finding is similar to those of previous studies showing that WFNS grades IV–V was correlated with a poor outcome and was a good predictor of unfavorable prognosis after aSAH (41, 42).

Furthermore, the DCI occurrence in the two cohorts was 35 and 15%, respectively. The onset of aSAH was concentrated during the COVID-19 period, which resulted in patients not receiving timely anti-vasospasm. It may be interpreted that the training cohort had a larger percentage of DCI. DCI was identified as another crucial prognostic factor in this study. The results of a large-scale retrospective study also suggested that DCI plays an important role in short-term and long-term prognosis (43). Cerebrovascular disorders, microthrombosis, cortical diffuse depolarization, and neuroinflammation all reportedly play potential roles in DCI pathogenesis (17, 44–46).

Contrary to nomograms or other models calculated as an approximation in previous studies, the web-based dynamic nomogram application developed in the present study (https://hu-ping.shinyapps.io/DynNomapp/) can be used to calculate an exact value for developing poor outcomes at 3 months after aSAH. This tool will help in the individualized treatment and management of patients, including neuro-intensive care, blood pressure control, nimodipine anti-vasospasm, anti-inflammatory drugs, and rehabilitation treatment, and help neurosurgeons make better clinical decisions.

However, this study has several limitations. First, this was a retrospective study; therefore, prospective studies are needed to verify our results. Second, owing to the low effective sample size in the training cohort, to reduce the data dimensionality and decrease multicollinearity between variables, we used LASSO regression analysis to select predictors. Third, future studies should collect complete clinical treatment information.

## Conclusion

This study identified independent predictors for an unfavorable prognosis in patients with aSAH, including NLR on admission, the WFNS grade, and DCI. On this basis, a web-based dynamic nomogram application, after externally validated, was developed to calculate the precise probability of a poor outcome in patients 3 months after aSAH. This will benefit personalized treatment and patient management and help neurosurgeons make better clinical decisions.

## Data Availability Statement

The raw data supporting the conclusions of this article will be made available by the authors, without undue reservation.

## Ethics Statement

The studies involving human participants were reviewed and approved by the Medical Ethics Committee of Renmin Hospital of Wuhan University. (approval number WDRM2021-K022). Written informed consent from the patients/participants or patients/participants legal guardian/next of kin was not required to participate in this study in accordance with the national legislation and the institutional requirements.

## Author Contributions

PH, YX, GD, and QC: Study design. PH, SZ, XZ, QS, and YW: Literature search. PH, YLiu, YLi, and LY: Data acquisition, data analysis, and statistical analysis. PH, YX, YQ, HZ, GD, and QC: Article preparation, editing, and review. All authors read and approved the final article.

## Conflict of Interest

The authors declare that the research was conducted in the absence of any commercial or financial relationships that could be construed as a potential conflict of interest.

## Publisher's Note

All claims expressed in this article are solely those of the authors and do not necessarily represent those of their affiliated organizations, or those of the publisher, the editors and the reviewers. Any product that may be evaluated in this article, or claim that may be made by its manufacturer, is not guaranteed or endorsed by the publisher.

## References

[1] Connolly ES Jr Rabinstein AA Carhuapoma JR Derdeyn CP Dion J Higashida RT. Guidelines for the management of aneurysmal subarachnoid hemorrhage: a guideline for healthcare professionals from the American Heart Association/american Stroke Association. Stroke. (2012) 43:1711–37. 10.1161/STR.0b013e3182587839 22556195

[2] Rinkel GJ Algra A. Long-term outcomes of patients with aneurysmal subarachnoid haemorrhage. Lancet Neurol. (2011) 10:349–56. 10.1016/S1474-4422(11)70017-5 21435599

[3] Mackey J Khoury JC Alwell K Moomaw CJ Kissela BM Flaherty ML. Stable incidence but declining case-fatality rates of subarachnoid hemorrhage in a population. Neurology. (2016) 87:2192–7. 10.1212/WNL.0000000000003353 27770074 PMC5123555

[4] Giede-Jeppe A Reichl J Sprügel MI Lücking H Hoelter P Eyüpoglu IY. Neutrophil-to-lymphocyte ratio as an independent predictor for unfavorable functional outcome in aneurysmal subarachnoid hemorrhage. J Neurosurg. (2019) 132:400–7. 10.3171/2018.9.JNS181975 30717052

[5] Zhang D Zhuang Z Wei Y Liu X Li W Gao Y. Association of admission serum glucose-phosphate ratio with severity and prognosis of aneurysmal subarachnoid hemorrhage. World Neurosurg. (2019) 127:e1145–51. 10.1016/j.wneu.2019.04.071 30995552

[6] Ma X Lan F Zhang Y. Associations between C-reactive protein and white blood cell count, occurrence of delayed cerebral ischemia and poor outcome following aneurysmal subarachnoid hemorrhage: a systematic review and meta-analysis. Acta Neurol Belg. (2021) 9:1–14. 10.1007/s13760-020-01496-y 33423218 PMC7796813

[7] Lai X Zhang W Ye M Liu X Luo X. Development and validation of a predictive model for the prognosis in aneurysmal subarachnoid hemorrhage. J Clin Lab Anal. (2020) 34:e23542. 10.1002/jcla.23542 32860455 PMC7755773

[8] Zhang P Li Y Zhang H Wang X Dong L Yan Z. Prognostic value of the systemic inflammation response index in patients with aneurismal subarachnoid hemorrhage and a Nomogram model construction. Br J Neurosurg. (2020) 12:1–7. 10.1080/02688697.2020.1831438 33044089

[9] Jaja BN Cusimano MD Etminan N Hanggi D Hasan D Ilodigwe D. Clinical prediction models for aneurysmal subarachnoid hemorrhage: a systematic review. Neurocrit Care. (2013) 18:143–53. 10.1007/s12028-012-9792-z 23138544

[10] Chen S Li X Lv H Wen X Ding Q Xue N. Prognostic dynamic nomogram integrated with inflammation-based factors for non-small cell lung cancer patients with chronic hepatitis B viral infection. Int J Biol Sci. (2018) 14:1813–21. 10.7150/ijbs.27260 30443185 PMC6231224

[11] Chen L Qian J Lin L Lin J Chen Q Zhuang Z. Prognostic value of preoperative lymphocyte-to-monocyte ratio in oral cancer patients and establishment of a dynamic nomogram. Oral Dis. (2021) 27:1127–36. 10.1111/odi.13629 32881142

[12] Bederson JB Connolly ES Jr Batjer HH Dacey RG Dion JE Diringer MN. Guidelines for the management of aneurysmal subarachnoid hemorrhage: a statement for healthcare professionals from a special writing group of the Stroke Council, American Heart Association. Stroke. (2009) 40:994–1025. 10.1161/STROKEAHA.108.191395 19164800

[13] Ajiboye N Chalouhi N Starke RM Zanaty M Bell R. Unruptured cerebral aneurysms: evaluation and management. Sci World J. (2015) 2015:954954. 10.1155/2015/954954 26146657 PMC4471401

[14] Frontera JA Claassen J Schmidt JM Wartenberg KE Temes R Connolly ES Jr. Prediction of symptomatic vasospasm after subarachnoid hemorrhage: the modified fisher scale. Neurosurgery. (2006) 59:21–7; discussion 21–7. 10.1227/01.NEU.0000218821.34014.1B 16823296

[15] Sano H Satoh A Murayama Y Kato Y Origasa H Inamasu J. Modified World Federation of Neurosurgical Societies subarachnoid hemorrhage grading system. World Neurosurg. (2015) 83:801–7. 10.1016/j.wneu.2014.12.032 25535068

[16] Zhang Y Zhu X Hou K Zhao J Gao X Sun Y. Clinical outcomes of surgical clipping for intracranial aneurysms in patients with a Hunt and Hess grade 4 or 5. Arq Neuropsiquiatr. (2016) 74:478–81. 10.1590/0004-282x20160064 27332073

[17] Vergouwen MD Vermeulen M Coert BA Stroes ES Roos YB. Microthrombosis after aneurysmal subarachnoid hemorrhage: an additional explanation for delayed cerebral ischemia. J Cereb Blood Flow Metab. (2008) 28:1761–70. 10.1038/jcbfm.2008.74 18628782

[18] Woo PYM Tse TPK Chan RSK Leung LNY Liu SKK Leung AYT. Computed tomography interobserver agreement in the assessment of aneurysmal subarachnoid hemorrhage and predictors for clinical outcome. J Neurointerv Surg. (2017) 9:1118–24. 10.1136/neurintsurg-2016-012576 29030464

[19] Vergouwen MD Vermeulen M Van Gijn J Rinkel GJ Wijdicks EF Muizelaar JP. Definition of delayed cerebral ischemia after aneurysmal subarachnoid hemorrhage as an outcome event in clinical trials and observational studies: proposal of a multidisciplinary research group. Stroke. (2010) 41:2391–5. 10.1161/STROKEAHA.110.589275 20798370

[20] Newcommon NJ Green TL Haley E Cooke T Hill MD. Improving the assessment of outcomes in stroke: use of a structured interview to assign grades on the modified Rankin Scale. Stroke. (2003) 34:377–8; author reply 377–8. 10.1161/01.STR.0000055766.99908.58 12574545

[21] Banks JL Marotta CA. Outcomes validity and reliability of the modified Rankin scale: implications for stroke clinical trials: a literature review and synthesis. Stroke. (2007) 38:1091–6. 10.1161/01.STR.0000258355.23810.c6 17272767

[22] Janssen PM Visser NA Dorhout Mees SM Klijn CJ Algra A Rinkel GJ. Comparison of telephone and face-to-face assessment of the modified Rankin Scale. Cerebrovasc Dis. (2010) 29:137–9. 10.1159/000262309 19955737

[23] Moons KG De Groot JA Bouwmeester W Vergouwe Y Mallett S Altman DG. Critical appraisal and data extraction for systematic reviews of prediction modelling studies: the CHARMS checklist. PLoS Med. (2014) 11:e1001744. 10.1371/journal.pmed.1001744 25314315 PMC4196729

[24] Pavlou M Ambler G Seaman S De Iorio M Omar RZ. Review and evaluation of penalised regression methods for risk prediction in low-dimensional data with few events. Stat Med. (2016) 35:1159–77. 10.1002/sim.6782 26514699 PMC4982098

[25] Steyerberg EW Eijkemans MJ Harrell FE Jr Habbema JD. Prognostic modeling with logistic regression analysis: in search of a sensible strategy in small data sets. Med Decis Making. (2001) 21:45–56. 10.1177/0272989X0102100106 11206946

[26] Sauerbrei W Royston P Binder H. Selection of important variables and determination of functional form for continuous predictors in multivariable model building. Stat Med. (2007) 26:5512–28. 10.1002/sim.3148 18058845

[27] Riley RD Snell KIE Martin GP Whittle R Archer L Sperrin M. Penalization and shrinkage methods produced unreliable clinical prediction models especially when sample size was small. J Clin Epidemiol. (2020) 132:88–96. 10.1016/j.jclinepi.2020.12.005 33307188 PMC8026952

[28] Hanley JA Mcneil BJ. The meaning and use of the area under a receiver operating characteristic (ROC) curve. Radiology. (1982) 143:29–36. 10.1148/radiology.143.1.7063747 7063747

[29] Vickers AJ Elkin EB. Decision curve analysis: a novel method for evaluating prediction models. Med Decis Making. (2006) 26:565–74. 10.1177/0272989X06295361 17099194 PMC2577036

[30] Van Calster B Wynants L Verbeek JFM Verbakel JY Christodoulou E Vickers AJ. Reporting and interpreting decision curve analysis: a guide for investigators. Eur Urol. (2018) 74:796–804. 10.1016/j.eururo.2018.08.038 30241973 PMC6261531

[31] Yan Y Hu J Fang X Zhen Y Feng L Zhang X. Predicting the poor recovery risk of aneurysmal subarachnoid hemorrhage: clinical evaluation and management based on a new predictive nomogram. Clin Neurol Neurosurg. (2021) 200:106302. 10.1016/j.clineuro.2020.106302 33092930

[32] Akkol S. The prediction of live weight of hair goats through penalized regression methods: LASSO and adaptive LASSO. Arch Anim Breed. (2018) 61:451–8. 10.5194/aab-61-451-2018 32175452 PMC7065407

[33] Guo R Wu Y Chen R Yu Z You C Ma L. Clinical value of neutrophil-to-lymphocyte ratio in primary intraventricular hemorrhage. World Neurosurg. (2019) 127:e1051–6. 10.1016/j.wneu.2019.04.040 30980971

[34] Wang JY Zhang XT Wang JQ Wang CY Zheng WL Pan ZM. Admission neutrophil-lymphocyte ratio predicts rebleeding following aneurismal subarachnoid hemorrhage. World Neurosurg. (2020) 138:e317–22. 10.1016/j.wneu.2020.02.112 32112936

[35] Lazaridis C Rusin CG Robertson CS. Secondary brain injury: predicting and preventing insults. Neuropharmacology. (2019) 145:145–52. 10.1016/j.neuropharm.2018.06.005 29885419

[36] Lattanzi S Di Napoli M Ricci S Divani AA. Matrix metalloproteinases in acute intracerebral hemorrhage. Neurotherapeutics. (2020) 17:484–96. 10.1007/s13311-020-00839-0 31975152 PMC7283398

[37] Zhou Y Wang Y Wang J Anne Stetler R Yang QW. Inflammation in intracerebral hemorrhage: from mechanisms to clinical translation. Prog Neurobiol. (2014) 115:25–44. 10.1016/j.pneurobio.2013.11.003 24291544

[38] Lattanzi S Brigo F Trinka E Cagnetti C Di Napoli M Silvestrini M. Neutrophil-to-lymphocyte ratio in acute cerebral hemorrhage: a system review. Transl Stroke Res. (2019) 10:137–45. 10.1007/s12975-018-0649-4 30090954

[39] Oshiro EM Walter KA Piantadosi S Witham TF Tamargo RJ. A new subarachnoid hemorrhage grading system based on the Glasgow Coma Scale: a comparison with the Hunt and Hess and World Federation of Neurological Surgeons Scales in a clinical series. Neurosurgery. (1997) 41:140–7; discussion 147–8. 10.1097/00006123-199707000-00029 9218306

[40] Jain S Iverson LM. Glasgow Coma Scale. In: Babak A. StatPearls. Treasure Island, FL: StatPearls Publishing Copyright, ©, 2020. StatPearls Publishing LLC. (2020).

[41] Zhao B Cao Y Tan X Zhao Y Wu J Zhong M. Complications and outcomes after early surgical treatment for poor-grade ruptured intracranial aneurysms: A multicenter retrospective cohort. Int J Surg. (2015) 23:57–61. 10.1016/j.ijsu.2015.09.008 26365431

[42] Shen J Huang K Shen J Zhu Y Jiang H Pan J. Clinical efficacy between microsurgical clipping and endovascular coiling in the treatment of ruptured poor-grade anterior circulation aneurysms. World Neurosurg. (2019) 127:e321–9. 10.1016/j.wneu.2019.02.248 30904812

[43] Olsen MH Orre M Leisner ACW Rasmussen R Bache S Welling KL. Delayed cerebral ischaemia in patients with aneurysmal subarachnoid haemorrhage: functional outcome and long-term mortality. Acta Anaesthesiol Scand. (2019) 63:1191–9. 10.1111/aas.13412 31173342

[44] Budohoski KP Guilfoyle M Helmy A Huuskonen T Czosnyka M Kirollos R. The pathophysiology and treatment of delayed cerebral ischaemia following subarachnoid haemorrhage. J Neurol Neurosurg Psychiatry. (2014) 85:1343–53. 10.1136/jnnp-2014-307711 24847164

[45] Engelhardt B Vajkoczy P Weller RO. The movers and shapers in immune privilege of the CNS. Nat Immunol. (2017) 18:123–31. 10.1038/ni.3666 28092374

[46] Meyer C Martin-Blondel G Liblau RS. Endothelial cells and lymphatics at the interface between the immune and central nervous systems: implications for multiple sclerosis. Curr Opin Neurol. (2017) 30:222–30. 10.1097/WCO.0000000000000454 28323646

